# Genetic Characterization and Alternative Preservation Ways of Locally Adapted Sheep Breeds: Cases of Private and Public Sheep Sectors in Tunisia and Italy

**DOI:** 10.3390/biology11111623

**Published:** 2022-11-06

**Authors:** Yousra Ben Sassi-Zaidy, Aziza Mohamed-Brahmi, Rafik Aloulou, Faouzia Charfi-Cheikhrouha, Filippo Cendron, Nicola Tormen, Martino Cassandro

**Affiliations:** 1 Laboratory of Diversity, Management and Conservation of Biological Systems, LR18ES06, Faculty of Sciences of Tunis, University of Tunis El Manar, Tunis 2092, Tunisia; 2 Department of Agronomy, Animal, Food, Natural Resources and Environment, University of Padova, 35020 Legnaro, Italy; 3 Laboratory of Management and Control of Animal and Environmental Resources in Semi-Arid Area, LR18AG01, Institut Supérieur Agronomique de Chott-Mariem, University of Sousse, ISA-CM-BP, 47, Chott-Mariem 4042, Tunisia; 4 Laboratory of Agricultural Production Systems Sustainability in the North Western Region of Tunisia, Department of Animal Production, Ecole Supérieure d’Agriculture du Kef Boulifa, University of Jendouba, Le Kef 7119, Tunisia; 5 National Federation of National Breeders Associations (FedANA), XXIV Maggio 43, 00187 Roma, Italy

**Keywords:** Mediterranean native sheep breeds, microsatellite markers, genetic diversity, preservation

## Abstract

**Simple Summary:**

The genetic characterization of native sheep breeds from the Tunisian-Italian Mediterranean corridor, the earliest westward introduction route of sheep breeding from the domestication center, was carried out using microsatellite markers in order to compare the genetic diversity level between both Mediterranean sides’ local sheep breeds, and to investigate their level of risk for conservation purposes. Sampling concerned both private and public/institutional farms of all the native Tunisian breeds in the case of the southern Mediterranean side, and one center Italian and the Venetian native breeds from the northern side. The results revealed that the genetic diversity level of the Tunisian native breeds was higher than that of the Italian ones, with a lower inbreeding level. The comparison between private and public farms in terms of genetic diversity, consanguinity, and conservation decisions highlighted the superiority of the public/institutional breeding management strategies over private ones in both Tunisian and Italian cases. The present study illustrated the efficiency of genomic characterization in making genetic diversity evaluations and preservation decisions of native and well-adapted sheep breeds in both developing and developed countries’ rearing conditions.

**Abstract:**

Non-commercialized sheep breeds known as local or native breeds are well adapted to their environmental constraints and constitute precious genetic resources that need prioritization for genetic diversity characterization and preservation. The aim of the present study was to assess the genetic diversity level and the related preservation decisions of very old and traditional native Mediterranean sheep breeds from Tunisia and Italy using 17 microsatellite markers. In total, 975 sheep were sampled from five Tunisian, one Center Italian, and four Venetian native breeds. Both private and publicly available farms were considered for each breed for breeding strategies’ comparison purposes. The microsatellite set used was highly informative (PIC = 0.80 ± 0.08), with a total of 383 alleles. Moderate genetic differentiation was revealed between the native sheep of the two Mediterranean sides (global overall loci F_ST_ = 0.081). The genetic diversity level was higher in the case of the Tunisian native breeds compared to the Italian ones, as evidenced by higher mean allelic richness, higher expected and observed heterozygosities, and lower inbreeding levels. Priority for conservation suggestions was carried out for each private or public breed population based on the contribution of each population to the diversity of the whole data. The four Venetian breeds, already undergoing conservation, the Tunisian dairy breed, and the very ancient Maghrebian breed, would be favored for conservation. In conclusion, our results highlighted the importance of the analyzed Mediterranean native sheep breeds as valuable inherited genetic reservoirs and supported previous conservation decisions made for the threatened breeds.

## 1. Introduction

Livestock diversity is crucial for food security, productivity and adaptability of production systems, resilience to climate change, and livelihoods [1]. The recognition of the local breeds’ contribution to fulfilling these functions has recently been endorsed [2,3]. In fact, locally adapted breeds developed in harsh production environments with poor feed quality, health problems, and extreme climatic conditions are expected to thrive and to cope with the climate change effects more easily than their modern counterparts, which struggle to survive in similar conditions [2]. Thus, the global action plan for animal genetic resources established by the FAO [4] to guide countries in improving the management of livestock genetic resources has prioritized local breeds that hold several adaptive characteristics, becoming more widely important [2]. This plan is based on four strategic priority areas, starting with the inventory, the characterization, and the monitoring of trends and associated risks of these animal genetic resources as the first strategic priority area, followed by their sustainable use, their conservation, and setting the necessary policies, institutions, and capacity-building to ensure these strategic priorities [3,4]. Despite the establishment of these priority areas since 2007, the FAO signaled in 2022 that 61.71% of local breeds are still being classified as “of Unknown Risk Status”, 27.77% as “At Risk”, and only 10.52% as “Not At Risk” [5]. The unknown risk classification reaches 84.34% in regions other than Europe, Caucasus, and North America, which indicates the necessity of improving knowledge and reporting the characteristics of local genetic resources to strengthen their sustainable management [1,6]. Concerning the situation of the Tunisian-Italian Mediterranean corridor, which includes several local breeds, since it was the earliest westward introduction route for domestic animals [1], the percentages of local breeds at risk are 90.27% in Italy and 17% in Tunisia, with the absence of unknown risk status local breeds in Italy versus 25% for the Tunisian ones [5]. Thus, further efforts are still needed for the assessment of the local breeds’ risk status in this narrow Mediterranean region.

The sheep genetic resources played an early major role in this Mediterranean corridor. The earliest westward introduction wave of sheep was mainly maritime and had reached the Mediterranean island regions between the 12th and 10th millennium BP [1,7]. The actual Corsican and Sardinian mouflons, which are the feral descendants of the first domestic immigrant sheep [7,8], are principal witnesses of this assumption. Accordingly, the local sheep breeds of this region, which have not undergone the strong selection of production traits, would have inherited a precious hot spot gene pool reservoir [9]. The assessment of the risk status, sustainable use, and conservation of these Mediterranean local sheep breeds is urgently needed. The last FAO statistics [5] declared that 86.89% of Italian local sheep breeds were classified as at risk. The FAO data of the local sheep resources in Tunisia have been recently updated and still need further editing to reflect the real situation and risk status of sheep resources. In fact, 40% of Tunisian local sheep are declared to be of unknown risk status, versus 40% are not at risk, and 20% are declared to be extinct, which is not the case because there is no extinct sheep breed in Tunisia. Currently, four local breeds were mentioned by the Domestic Animal Diversity Information System (DAD-IS) [10]: the “Barbarine” employed to designate the unique fat-tailed Tunisian sheep breed, which is classified as a transboundary breed, is without risk status; the “Noire de Thibar” employed to designate the black thin-tailed breed created in the Northern-West “Thibar” region of Tunisia [11], has no clear risk status (declared simultaneously “with unknown risk status” and “not at risk”); the “Sicilo-Sarde” employed to designate the dairy breed which is declared to be with unknown risk status; and, the “Queue Fine de l’Ouest” employed to designate the white thin-tailed breed of the western Tunisian border, declared as “not at risk”. The D’man breed, introduced since the nineties of the last century from the Moroccan oasis to populate the oasis breeding system of Tunisia, is currently reared even in central and northern Tunisian intensive breeding systems [12] and presents well adaptive traits under relative fluctuant climatic conditions. It was recently proved by archeological and molecular genetic data that the D’man breed was reared in Tunisia and all the Maghreb region in the Neolithic [13]. The import of this breed from Morocco might be considered a reintroduction, which justifies its good adaptability to Tunisian conditions. This breed is considered a native Maghrebian breed common to Morocco, Algeria, and Tunisia. Actually, the identification and genetic diversity threats’ assessment of Tunisian sheep resources need to be improved. The characterization, conservation, and sustainable management of ancient local sheep genetic resources in the Mediterranean Tunisian-Italian corridor, the earliest Neolithic cradle of western Mediterranean sheep breeding [13], is still an urgent challenging task to preserve their precious gene pool reservoir. Thus, the objective of this study was to characterize the genetic diversity and the relative risk status of the total Tunisian sheep breeds, and five very ancient Italian local sheep breeds using genomic data based on microsatellite markers. Both private and public farms were considered in this study in order to assess the effect of breeding management on the genetic diversity level.

## 2. Materials and Methods

### 2.1. Sample Collection and Description

A total of 975 blood samples were collected from Tunisian and Italian sheep by specialized veterinarians of the local health authorities during sanitary control from both private and, if available, public/institutional farms for each breed. According to the Research Ethics Committee of Padova University, ethical review and approval were waived for this study, and no specific authorization was required. No more than four unrelated animals were sampled from each flock based on the information provided by farmers and breeders when pedigree data were not available. An entire number of 249 individuals were sampled from Tunisia, belonging to the five Tunisian locally adapted breeds: Barbarine (BAR, *n* = 64), Queue Fine de l’Ouest (QFO), *n* = 41), Noire de Thibar (NTH, *n* = 41), Sicilo-Sarde (SS, *n* = 45), and the D’man breed (DM, *n* = 28). A recently created Tunisian crossbred BAR × QFO (CRO, *n* = 30) was also investigated to assess the effect of this new breeding trend on the genetic variability level of Tunisian sheep resources. A total of 695 individuals belong to the four Venetian local breeds: Lamon (LAM, *n* = 141), Alpagota (ALP, *n* = 250), Brogna (BRO, *n* = 186), and Foza named likewise Vicentina (FOZ, *n* = 118). A sample of 31 individuals belonged to the Central-Italian Appenninica native breed (APP, *n* = 31). Although the analyzed Italian breeds are strictly defined based on a set of phenotypic standards with breed herd books and formalized breeds’ associations [14], the analyzed Tunisian breeds are mainly identified and maintained by breeders as separate breeds based on phenotypic characteristics and geographic localities. This breed classification was adopted by government authorities and supported by scientific research that has justified these Tunisian breeds’ classification [15,16]. The choice of the analyzed Italian native breeds is based on their common morphological and genetic background shared with the Tunisian breeds. This very ancient relatedness was recently explored by Ben Sassi-Zaidy et al. [13]. All the Tunisian and Italian sampled breeds are thin-tailed except the BAR, which is the unique West Mediterranean fat-tailed breed assumed as the ancestor of the Italian Barbaresca and Laticauda Italian breeds [8,17]. Both private and publicly available farms were considered in the case of each breed for breeding strategies’ comparison purposes. The Tunisian sampled public farms included in this study belonged to the OTD (Office des Terres Domaniales: State Lands Office), founded in 1961. This office currently holds 97 flocks with about 55,000 sheep heads and 17,000 female units, including 3 local meat breeds (BAR, QFO, and NTH) and one dairy breed, the SS [18,19]. Concerning the Italian breeds, the public farm included in this study is the “Villiago” Conservation Center of the Venetian Mountain, which is an institutional experimental farm holding the four endangered Venetian native breeds LAM, ALP, BRO, and FOZ, undergoing an in situ conservation program. Further details about the sampled breeds are summarized in Table 1.

### 2.2. DNA Extraction and Genotyping

Genomic DNA extraction was carried out from 200 µL whole blood using the Wizard Genomic DNA Extraction Kit (Promega, Madison, WI, USA) following the manufacturer’s protocol. The total of samples were genotyped at 17 microsatellite loci panel (Table 2) established from the ISAG-recommended microsatellite markers [22] and from previous sheep genetic diversity studies in the Mediterranean region [21,23,24,25]. Genotypes for all 17 microsatellite markers were determined by means of three multiplex PCR reactions using fluorescence-labeled primers in a total volume of 12.5 µL. Amplification was performed using standard PCR reactions in a GeneAmp 9700 thermal cycler (Life Technologies, Carlsbad, CA, USA), starting with 50 ng of purified DNA. The 17 microsatellites were amplified with the following conditions: initial denaturation step of 5 min at 95 °C, 35 cycles of 30 s at 95 °C, 1 min 30 s at 61 °C and 30 s at 72 °C and a final extension of 30 min at 60 °C. Multiplexes pooled by capillary electrophoresis and allele size were performed with a CEQ 8000 Genetic Analysis System (Beckman Coulter, Fullerton, CA, USA).

### 2.3. Statistical Analysis

The MICRO-CHECKER version 2.2.3 [26] software was used to assess genotyping quality, occurrence of null alleles, stuttering, and allelic dropout. Exact tests for deviations from Hardy–Weinberg Equilibrium (HWE) and linkage disequilibrium (LD) were applied using GENEPOP version 4.3 [27]. The number of alleles per locus (NA), allelic frequencies, observed (H_o_) and expected (H_e_) heterozygosities were calculated using GENETIX version 4.05.2 [28]. The number of private alleles (P_AR_) in the different populations using the hierarchical rarefaction method was counted using HP-RARE software [29]. MSA software [30] was used to calculate allelic richness (AR, the mean number of alleles per locus corrected by sample size), Wright’s fixation indices (F_IS_, F_IT_, and F_ST_) [31] and the pair-wise population Proportion of Shared Alleles (POSA) distances matrix. The Polymorphic Information Content (PIC) was measured using MOLKIN 3.0 [32]. This software was likewise used to set conservation priorities for the analyzed subpopulations based on the contribution of each analyzed population to the global diversity of the metapopulation, as defined in the Caballero and Toro method [33]. The global value (Loss (−) or Gain (+)) of genetic diversity (GD in %) after the hypothetic removal of each analyzed sheep subpopulation constitutes its contribution to the overall genetic diversity and is considered for making conservation priorities. Subpopulations presenting a loss of GD (negative value) after their assumed removal are a priority for conservation decisions. The global GD value of each subpopulation results from the balance between its internal diversity value and its mean distance value from the remaining subpopulations (a measure of the between-subpopulation diversity) [33]. GENETIX version 4.03 [28] was used to perform the Factorial Correspondence Analysis (FCA) based on individual multilocus genotypes. The neighbor network of the analyzed breeds was built from pair-wise POSA distances using the Splits Tree 5 program [34]. The assessment of the differentiation degree and the population structure of the investigated breeds was performed using the Bayesian clustering approach implemented in STRUCTURE software v.2.3.4 [35] To choose the appropriate number of inferred clusters to model the data, 50 independent runs were performed for each K cluster value (2 < K < 12). All analyses used a burn-in period of 50,000 and 150,000 iterations for data collection. The optimum number of clusters fitting the data was established following the Δ K Evanno’s method [36] using the STRUCTURE HARVESTER program [37]. The output obtained was used directly as input data in the cluster visualization program CLUMPAK [38].

## 3. Results and Discussion

### 3.1. Microsatellite Panel Variability

All 17 microsatellite markers spread over 14 ovine chromosomes have been passed on the quality control settings and were considered in the genotyping data. A summary of the polymorphism of the genotyped microsatellite panel in the Tunisian local breeds, in the Italian local breeds, and in the combined whole dataset is presented in Table 2. A total of 383 alleles were detected in the whole dataset, with a mean number of 22.53 ± 6.07 alleles, which ranged from 17.47 ± 5.75 detected in the Tunisian breeds to 20.06 ± 5.25 in the Italian ones. Eliminating the population size bias, the AR of the Tunisian breeds was higher than that of the Italian one (10.70 ± 3.07 and 7.63 ± 1.49, respectively). All of the microsatellite markers were highly informative, having an average high PIC > 0.5 (0.80 ± 0.08), with 81% in the Tunisian breeds and 78.8% in the Italian breeds investigated. The OarAE119 marker is more polymorphic in the Tunisian breeds (PIC = 0.81) than in the Italian one (PIC = 0.66), contrary to the OarAE129 (PIC = 0.60 and 0.84 for the Tunisian and Italian breeds, respectively). Considering the overall population, the mean values of the Wright’s Fixation indices F_IS_, F_IT_ and F_ST_ were 0.109, 0.180, and 0.081, respectively (*p* < 0.001). These values explained that the global homozygote excess (F_IT_) of 18% was mainly due to the within-breed homozygote excess (F_IS_) of 10.9%, indicating a notable inbreeding level among and within breeds. The genetic differentiation between breeds limited to 8.1% highlighted the moderate distinctiveness between the analyzed breeds, despite their belonging to different Mediterranean shores. This moderate differentiation level could be explained by the shared historical sheep migration events of the Tunisian-Italian corridor, beginning since the Neolithic and continuing in Carthaginian and Roman times. The between-breed differentiation levels, divergence times, and gene flow were detailed by Ben Sassi-Zaidy et al. [13]. Consequently, because of this moderate variation among breeds, a large part of the total genetic variability (82%) can be explained by the between-individual variability. The high number of alleles for each locus (16 to 38), as well as the PIC values (higher than 0.5), confirmed that all microsatellite markers used in this study were appropriate to evaluate the biodiversity of the Tunisian-Italian local sheep breeds, as well as proved by previous studies on native sheep breeds [17,21,23,25]. Microsatellites are the most frequently used markers for genotyping local farm animal breeds between 2005 and 2020 [39]. Although they are gradually being replaced by single nucleotide polymorphism (SNP) markers, the microsatellite markers are still used to investigate the genetic diversity of local sheep breeds [40,41,42,43], since commercial SNPs’ high throughput have been designed based on commercial breeds and thus, these markers presented an ascertainment bias regarding the local breeds [44]. Furthermore, the major shortcomings of the microsatellite markers, which is the challenge of genotyping repeatability across laboratories for dataset combination between different breed origins [45], was avoided in our study because the genotyping of the whole data from the two countries was conducted in the same laboratory.

### 3.2. Breed Variability

The genetic variability of each analyzed sheep breed was revealed in terms of (AR), private allelic richness (P_AR_), observed (Ho) and expected (He) heterozygosities, within-breed homozygote excess (F_IS_), and number of loci deviated from Hardy–Weinberg equilibrium (HWE), as shown in Table 3. The Tunisian sheep breeds were more genetically diverse at the 17 loci than the Italian local breeds, as evident from the high allelic diversity (AR varying from 9.29 ± 2.34 to 10.40 ± 3.05 in Tunisian breeds versus 7.56 ± 2.41 to 9.01 ± 1.73 in Italian breeds) and gene diversity (He varying from 0.80 ± 0.01 to 0.83 ± 0.10 in Tunisian breeds versus 0.76 ± 0.08 to 0.81 ± 0.09 in Italian breeds). The P_AR_ varied from 0.16 to 0.45 in the Tunisian breeds and from 0.21 to 0.38 in the Italian breeds. High frequencies are related to the breeds’ specificities in the two countries. Both Mediterranean sides’ local breeds showed considerable Ho values but were always lower than He. The deviation from HWE did not exceed the two loci in the Tunisian breeds group. However, in the Italian group, the number of loci in disequilibrium varied from 03 in APP to 12 in ALP (*p* < 0.001). Excluding null alleles’ cause after specific test control, the population substructure could explain this divergence, since the inbreeding level of the Italian local breeds was not that high (F_IS_ varying from 0.101 ± 0.101 in LAM to 0.134 ± 0.132 in ALP) and was not proportional to the number of deviated loci. This structuration within Venetian breeds has already been highlighted [13,21]. Similar F_IS_ values were observed for the Tunisian breeds except the NTH breed and the BAR unique fat-tailed breed, which presented the lowest inbreeding level (0.071 and 0.073, respectively).

The contribution of each Tunisian and Italian breed to the overall Tunisian-Italian sheep genetic diversity and the conservation decision’s proposition based on the loss or gain of genetic diversity (GD in %) after the hypothetical removal of each breed, as defined in Caballero and Toro’s approach [33], are illustrated in Table 3. Only the removal of the SS and DM Tunisian breeds from the whole data caused, respectively, a quite low loss of −0.06% and −0.04% of the total genetic diversity of the analyzed sheep resources. In the case of Italian sheep resources, no genetic diversity loss was revealed after the removal of the center Italian APP breed. The removal of each Venetian local breed resulted in a loss of genetic diversity ranging from −0.49% to −0.28%. The efficiency of the Caballero and Toro’s approach was also revealed in other livestock species, such as the Guzera Zebu cattle breed [46], in which lower percentages of genetic diversity loss ranging from −0.26% to −0.002% were highlighted after the removal of different lineages of this cattle breed.

The revealed difference in the genetic architecture between the Tunisian and the Italian local sheep resources would reflect the difference in the environment and the breeding practices between the two countries, especially since the beginning of sheep breeding industrialization in Europe, which has even influenced the local breeds. In fact, the focus on highly productive and selected cosmopolitan breeds in Italy has caused the native breed’s negligence. Consequently, a drastic decrease in the local Venetian sheep population has occurred [21,47], leading to the detected low genetic variability level of the four endangered Venetian breeds undergoing conservation strategies. The situation is not at risk for the APP native breed, even appearing as the lowest genetically variable within the Italian-analyzed breeds. However, the considerable genetic variability level of APP compared to other Italian breeds has been proven by several studies [48,49]. This difference in the variability level would be proportional to the pressure degree of the production traits’ selection applied to the analyzed Italian breeds. The highest genetic variability level observed within the Tunisian breeds would be related to the traditional sheep breeding system maintained in the country until the last decades of the 20th century. This traditional livestock system was based on a pastoral society rearing native and locally adapted breeds that had been empirically selected to address adaptability and resilience to harsh conditions rather than high productivity [50,51]. Consequently, Tunisian genetic resources appeared to be less influenced by modern breeding techniques, thus preserving a large part of their inherited genetic variability reservoir.

### 3.3. Issues of Genetic Variability Analysis and Relative Conservation Decisions

Measuring genetic diversity in locally adapted and native sheep animal breeds using neutral and maternal molecular markers is essential to setting up conservation strategies [39,52,53,54]. In fact, filling the knowledge gap on the genomic characterization of sheep resources is a prerequisite for the effective implementation of genetic improvement programs, especially in developing countries’ conditions [55], such as in the Tunisian case. The final decisions to prioritize different breeds for sustainable management programs and conservation should mainly be based on genetic variability information in addition to adaptability and sociocultural and economic factors [33]. The issues of the present genetic variability investigation of the analyzed Tunisian and Italian sheep genetic resources should support the establishment and the update of the management and conservation programs. These programs should strive to retain their genetic variability and to increase their population size, especially in the case of endangered breeds with small population sizes, taking into account several factors, such as their adaptability, economic, or scientific traits, as well as their historical and cultural values [56]. These overall factors will be discussed later separately for the Tunisian and Italian analyzed sheep resources.

#### 3.3.1. Issue of the Genetic Variability Analysis: Case of Tunisian Local Sheep Breeds

Tunisian sheep genetic resources are mostly formed by more or less ancient breeds that have not been subjected to the same strong selective forces as industrialized breeds in developed countries. The management of these breeds is 80% held by private breeders, and 70% of these are small breeders (<11 breeding ewes) who have inherited this activity from father to son without known management programs [20,57]. This major part of Tunisian sheep resources has mainly resulted from natural selection and adaptation, as well as empirical and low selection pressure on livelihood production traits linked to local traditions [15,58]. The public sector represents 20% of the Tunisian sheep breeding system. This sector is still managed by the Livestock and Pastures Office (OEP), which monitors a genetic improvement program initiated in the early seventies of the last century. The performance-recording schema within selection-base flocks has been the major component of the Tunisian national genetic improvement program, starting with the BAR breed and encompassing, thereafter, the QFO and NTH meat breeds and the SS dairy breed. Within the selection-base herds, the OEP carried out the improvement schema activities, including identification, performance recording, evaluation, selection of replacement females, and improved young rams, as well as culling activity [58]. The most significant aspect of the breeds’ genetic improvement program is the selection by the OEP of the best-performing lambs from the selection base and the production of improved rams [58,59]. Currently, the strategies are focused on the improvement of meat production capacities of the BAR, QFO, and NTH local meat breeds through the recording of the lambs’ growth performances. Since the severe decline in the number of dairy SS flocks, the milk-recording schema has been disturbed, leading to discontinuity in the SS’s milk production performance improvement program [58,59]. Despite the establishment of this national genetic improvement program since the 1970s in the public sheep-breeding sector, the private sector-controlled herds’ rate has not exceeded 15% in 2022, with only 8.42% of identified ewes and 7% of controlled lambs. This low intervention percentage is related to public financial limits and to the low breeders’ membership rate to pay identification and control services fees [59]. The major part of the publicly controlled sheep (56%) currently belong to the OTD, with 69 controlled flocks composed of 17,804 ewes and 14,561 lambs [19,59,60].

Considering both private and public sheep breeds as the overall Tunisian genetic sheep resources data, the genetic variability assessment in our study revealed the BAR fat-tailed breed as the most variable, presenting the highest expected and observed heterozygosities, a low homozygote excess coefficient, a high level of private allele richness, and the highest contribution to the overall genetic diversity of the Tunisian-Italian (+0.23%) and the Tunisian (+0.52%, data not shown in Table 3) pools. In addition, the greatest loss of internal diversity was obtained by the removal of BAR from the Tunisian-Italian pool (−0.59%, data not shown in Table 3). The lowest homozygotes level in BAR could be explained by the paternity control system practiced by shepherds, who assist the ewes during mating to lift the large fat tail, which represents a natural obstacle to free mating [61]. This practice was conferring to breeders a paternity control, since mating between close relatives is generally avoided [17] decreasing the inbreeding level. A lower average inbreeding coefficient value (0.058 ± 0.133) was revealed by analyzing all the BAR ecotypes reared in different Tunisian climate stages [17]. The fat-tailed BAR breed retains important sociocultural and historical values. It was presented as the most frequent symbol on the Carthaginian monuments [62], and the most current preferred breed in religious and familial ceremonies. This breed is characterized by its high rusticity and strong adaptability to both hot and cold climates. The BAR breed is also characterized by its high maternal abilities, resistance to internal and external parasites, ability to use low-quality feed resources and ability to deposit and mobilize body reserves from the fat tail and the rest of the body, and to remain productive under harsh conditions [61,63,64,65,66]. The BAR breed, currently counting 6,531,400 heads, includes only 50,000 pure bred animals that are reared within large-sized flocks (>250 heads) belonging to the public sector and enrolled in the herd book [5]. Private and public BAR flocks are raised in all Tunisian climatic systems from north to south, with a concentration in the central semi-arid and southern arid ecosystems [61]. The BAR management system was extensive. Currently, semi-intensive breeding systems are expanding, and four livestock systems are identified [67]; three of them are based on the raising of ewes and lamb production, under transhumance pastoralism or crop-livestock system or multi-active families’ system. The fourth system, which is developing strongly, is the “trader-fattener” indoor system based on fattening purchased lean lambs with a grain or concentrate regimen, without raising ewes. The first three breeding systems maintained, until the last few decades, the inherited traditional pastoralism knowledge preserving the BAR’s gene pool reservoir during many millennium [20,68]. In fact, traditional breeders or shepherds have preserved traditional local knowledge and skills of herd management, especially mating assistance, to lift the fat tail. The current unavailability of shepherds constitutes a main threat to BAR breeding, and this traditional knowledge is at risk of disappearing. In general, the three systems of raising ewes and lambs encounter diverse threats and are decreasing. Many farmers think of giving up sheep breeding [50,67]. Mixed BAR-QFO flocks are currently increasing in sheep breeding practices, and anarchical crossbreeding trends between these two breeds are frequently expanding in all Tunisian breeding systems, especially in the Center and the North [24,58]. Considered as a superior meat breed with an historic socio-cultural importance, the taste quality of the BAR breed meat needs to be further highlighted, as strongly proven in the BAR’s originated USA Tunis breed carcass, described as high-quality carcasses with excellent meat-to-bone ratios [69]. These superior BARs’ traits would probably be reflected in the considerable richness in private alleles (34%), which reached 52% in a previous study of local Tunisian sheep [70]. In a survey made by Djemali at al. [71], the preference for BAR’s meat due to its good quality is signaled in 59% of the consumers’ breed preferences’ reasons. The farmer’s preference for BAR breed over other breeds is due to its hardiness in 88% of their responses, and due to its meat quality in 54%, versus 1% and 17% of these traits, respectively, for the QFO breed. These unique traits and the high genetic variability level of BAR are threatened to be diluted and absorbed by an anarchic crossing with the QFO breed, creating recently (in the few last decades) the CRO crossbred population exhibiting low allelic diversity (AR = 09.73 ± 3.01), the lowest genetic diversity (He = 0.80 ± 0.10 and Ho = 0.68 ± 0.17), and the highest F_IS_ value (0.139 ± 0.192). The excess homozygotes in this population could be explained by the anarchic crosses practiced without any parentage control, as declared by the breeders who frequently practice this cross between BAR males and QFO females to obtain a smaller tail, thus missing the paternity control present in BAR mating management, since the thin tail of the QFO breed does not require the shepherd assistance in the mating. This anarchic crossing was imposed by butchers, who found it difficult to sell the heavy-weight fat of the BAR tail [64,71]. The genetic erosion and dilution of the BAR’s gene pool following this practice is clearly highlighted by the structure analysis computed by Ben Sassi-Zaidy et al. [24] encompassing the three partners of this crossing. The same genetic absorption problem of many local Algerian breeds [72,73] and even Moroccan local sheep breeds [74] by crossing with the Algerian Ouled Djellal breed has spread to the West into Morocco and to the East into Tunisia, giving the QFO Tunisian breed. These trends constitute a real threat to local genetic sheep resources in the Maghreb region. The FAO considers that the erosion of within-breed diversity can be a problem, even in breeds whose total population size remains large [1]. In fact, in Tunisia, even though the BAR remains the most dominant breed, its spatial distribution and its presence percentage are decreasing in favor of the QFO (from 85% in the 1970s to 60.3% in 2011 and 58% in 2015 for the BAR versus 9% in the 1970s to 34.6% in 2011 to 37% in 2015 for QFO) [58,75].

As mentioned above, the QFO breed is assumed to originate from Algerian Ouled Djellal sheep. However, this breed is considered indigenous to the western highlands of Tunisia [58]. Until now, no study has included the two neighboring breeds, QFO and Ouled Djellal, to assess their differentiation levels. The population size of the QFO is about 1,216,112 heads, representing 35% of the total sheep resources [5]. Rekik et al. [58] mentioned that the QFO is most commonly raised under an agrosylvo-pastoral system in the highlands within small- to medium-sized flocks (<50 heads) owned by private sedentary farmers combining cereal cropping with sheep production. A new form of peri-urban systems is emerging in which lamb fattening is an important activity. This production system is encouraged by access to secondary cereals and more extensive marketing opportunities in urban areas. Consequently, since the end of the last century, the QFO has gradually invaded all Tunisian territory, especially in the last two decades, where mixed BAR-QFO flocks are emerging in agro-pastoral and peri-urban zones, although not well documented [20]. As declared by breeders (mainly the youngest ones), avoiding the shepherd assistance during mating and responding to the butcher exigency for thin tail or intermediate tail (of the CRO population) due to the difficulty of selling the fat of the BAR tail that is no longer asked for by consumers are the main causes of the QFO introduction in the BAR flocks. The oldest breeders, mainly in the arid pastoral systems in the southern Tunisian regions, still prefer BAR breeding despite its more complicated management knowledge and skills that they have inherited through several generations of pastoralism [68], thus due to its hardiness and excellent adaptability traits. Further scientific analysis is needed to well document these trends and to maintain the traditional knowledge of the BAR breeding practices as declared in the Nagoya protocol and strongly required by the FAO [1]. Based on the current genetic diversity investigation of the QFO, the highest AR (10.40 ± 3.05) noted in the breed reflected the introduction of permanent new alleles. The considerable genetic diversity level of the QFO (Table 3), as well as its adaptability to the highlands of the western Tunisia area and its good walking ability, let us consider this breed as a valuable genetic resource. In fact, the removal of the QFO from the Mediterranean pool resulted in a loss of within-breed diversity of −0.49% (second range after the BAR (−0.59%), data not shown in Table 3). However, the absorption of the BAR’s gene pool by its crossing with the QFO requires urgent political decisions that must be taken to avoid uncontrolled crossing of this last breed with other local Tunisian sheep resources to preserve their integrity and purity.

The NTH breed, currently counting about 76,000 ewes (2% of the total sheep resources), was created in the Thibar region at the beginning of the 1900s’ through a crossbreeding schema between the Algerian Ouled Djellal breed and the French Merinos. The selection was primarily placed to fix the black color by highly inbred mating series to avoid the white sheep photosensitization engendered by the consumption of the *Hypericum perforatum* weed growing with cereal crops in northern sheep breeding systems [15,17]. After the black color fixation (from which appeared the name “Noire de Thibar,” meaning Black of Thibar), selection in the 1960s has been applied for many traits, such as wool and lamb growth rate and reproduction performances, and after that, it has been entirely applied for growth performances [15,58]. The NTH management system is mainly semi-intensive in large private farms, cooperatives, or public farms. This system is based on flock grazing for cereal production, and afterwards the use of produced complementary forages such as hay and silage. Despite the inbreeding problems previously reported within the breed [11,58], the NTH showed a high genetic variability level with high He, Ho, and lowest heterozygotes deficit level (F_IS_ = 0.071 ± 0.121). This could be explained by the strong efforts made to reduce the impact of inbreeding on NTH [11,76]. In fact, the introduction of rams’ genes from the Brown Swiss breed by artificial insemination [77] and Portuguese Black Merino breed to the named “Sidi Tabet Cross” flock [11] seems to have a good part in reversing the problem of inbreeding in the NTH. Currently, this breed is mostly reared in the northwest on big farms with expert management, leading to the resolution of consanguinity and low production performance. In fact, as mentioned by Chalh et al. [11], the NTH low production could be attributed to non-genetic factors. Except for its resistance to the phototoxic effect, the NTH meat breed does not present specific traits, which can probably be explained by the smallest private alleles’ richness of this breed (16%). In fact, the NTH meat quality does not exceed 5% of the consumers preference [71]. Based on Caballero and Toro’s method (Table 3), the hypothetic removal of the NTH will not reduce the overall Tunisian-Italian pool genetic diversity. Nevertheless, considering only the Tunisian breeds as the overall genetic pool (data not shown in Table 3), the NTH removal contributed to a loss of 0.11% of the global Tunisian sheep diversity, which resulted from an increase of the overall pool diversity of +0.12% due to its internal diversity, and a reduction of −0.23% due to its negative mean distance from the remaining breeds. In fact, the assumed removal of the NTH will reduce the overall Tunisian sheep variability, which led to the application of a conservation decision to preserve this native breed well adapted to its local production system.

The SS is a unique North African dairy breed created early in the last century from a crossbreeding schema between the Italian Sarda and Comisana dairy breeds in the northern region of Tunisia [25]. Counting about 38,000 ewes, the SS represents 1% of the total Tunisian sheep resources [78]. This breed, reared to produce high-quality milk and typical traditional cheese, is mostly found in the sub-humid Beja and Mateur regions due to its lesser adaptability to the harsh conditions of semi-arid production systems [15,25]. It is raised under two different production systems: a semi-extensive system in private or cooperative farms with large flocks (up to 200 breeding ewes) based on flock integration with cereal and forage production, and an extensive system in the mountains and foothills with small flocks less than 20 head [58]. The SS is mainly managed as a dual-purpose (milk and meat) breed, applying a long suckling period of more than 3 months to obtain heavier lambs [79,80]. Despite this practice, SS lambs present the lowest growth performance among Tunisian breeds [58]. The dairy specificity of SS is reflected by its high richness in private alleles (38%). This richness reached 67%, analyzing only local Tunisian breeds [70]. Although this breed has overcome the bottleneck effect due to its last dramatic population decline [25,81,82], its within and between subpopulation variability balance based on Caballero and Toro’s method (Table 3) revealed its priority for conservation decisions. In fact, the hypothetic extinction of the SS breed resulted in a reduction of the internal breed diversity of −0.36% from the Tunisian-Italian pool and of −0.046% from the Tunisian sheep diversity pool, respectively, and into a global GD loss of −0.064% and −0.47% from these two pools, respectively (data not shown in Table 3). Accordingly, more adequate conservation decisions need to be made to conserve this unique Tunisian local dairy sheep resource as superior as highly specialized Mediterranean dairy sheep [25]. The “Unknown” risk status of the SS breed declared by the FAO (DAD-IS) [10] should therefore be changed to “At Risk” status.

The DM breed, considered until recently an exotic breed introduced to Tunisia from Morocco, is the remaining form of ancestral sheep reaching Tunisia and all the North African land in the Neolithic [13]. This prolific breed, presenting a precocious puberty and a continuous breeding season, was mainly raised under intensive management in Tunisian oases in an accelerated lambing system, with three lambings every two years [83]. Lambs, mostly triplets and more, were suckled by their mothers until weaning occurred after 2 months. Supplementary suckling is usually needed to help weak lambs [83]. The population size of DM represents less than 1%, with about 5000 ewes [78]. Our results revealed that the DM presented a high allelic diversity level with the highest richness in specific alleles of all the analyzed Mediterranean breeds. The gene diversity level of this breed was considerable (He = 0.82 ± 0.01). The lack of heterozygotes revealed in DM (0.123 ± 0.138) could explain the consanguinity problem reported in this breed, which was reintroduced in Tunisia in the 1990’s from Morocco as only 200 ewes and 12 rams, and reared in the isolated oasis areas without genetic exchange with other breeds [77]. According to Caballero and Toro’s approach results (Table 3), the conservation of the DM needs to be undertaken since it presents a precious very old gene pool heritage [13]. The assumed removal of the DM breed from the Tunisian pool (data not shown in Table 3) resulted in the greatest global GD loss (−0.48%) due to the greatest loss of divergence from the remaining breeds (mean distance from breeds of −0.51%).

As mentioned by Caballero and Toro [33], maximization of genetic diversity will lead to maximum allelic richness in long-term conservation programs and will be appropriate as a general guide for conservation. Applying Caballero and Toro’s approach [33] to the local Tunisian sheep genetic diversity as the whole genetic diversity data (result not shown in Table 3), only the removal of the fat-tailed BAR breed will not reduce the overall diversity of the Tunisian sheep pool. This result is due to its highest internal diversity (+0.23%) and highest mean distance from the remaining breeds (+0.29%), with a global GD gain of +0.52%. The removal of the remaining Tunisian thin-tailed breeds NTH, QFO, SS, and DM resulted in a global loss of the GD of −0.11%, −0.13%, −0.47% and −0.48% respectively. Adequate conservation decisions need to be taken to conserve these native and locally adapted Tunisian genetic resources, especially for the SS as the unique south Mediterranean dairy breed, and the very old and prolific North African DM breed that constitutes a very old and genetic sheep heritage and is well adapted to the warm and dry Saharan climate.

In general, the genetic diversity (He) of all the local Tunisian breeds, except the CRO population, is considerable and can be oriented toward short-term and long-term conservation strategies. Coherent in situ conservation strategies based on the avoidance of inbred mating and breeds’ erosion seem to be urgently needed for locally adaptive Tunisian breeds’ conservation.

#### 3.3.2. Issue of the Genetic Variability Analysis: Case of the Italian Local Sheep Breeds

Italian local sheep genetic resources are almost 87% formed by breeds classified as at risk [5]. In the Veneto Region, the north-eastern part of Italy, several native breeds have disappeared [84] and the sheep population decreased dramatically from 101,170 animals in 1953 to 34,734 animals in 1991 [85]. Currently, the total number of Venetian native sheep is estimated at only 5300 heads represented by four native breeds that are still reared in the Veneto region: ALP, BRO, FOZ, and LAM. These breeds, classified by the FAO (DAD-IS) as “at risk” [10], have been reared for many centuries under two different breeding systems:

- The transhumant system, mainly used for the large-sized and long-legged FOZ and LAM breeds, reared with large flocks grazing on plains and coastal areas during winter, and displacing to highland pastures in summer,
- The mountainous grassland-based farming livestock system, known as small flocks reared on farms located in mountainous and hilly areas, is mainly used for the two medium-sized ALP and BRO breeds [47,86].

The detailed local risk status declared by the FAO (DAD-IS) [10] is “Critical” for LAM and FOZ breeds that are considered at risk of extinction because of their small population size of 385 and 197 animals, respectively [10]. The situation of ALP and BRO is less critical, with larger current population sizes reaching 1923 and 2802 heads in 2020, respectively, thus presenting as “Endangered” detailed local risk status declared by FAO (DAD-IS) [10]. Due to the good adaptive traits of these threatened Venetian sheep breeds, their environmental role in exploiting marginal pastures, and their participation in the socio-cultural preservation of traditions and cultural heritage, these breeds represent an important reservoir of genetic resources that need to be conserved [21,47,86]. Several efforts have been made since the last decade to conserve these native Venetian breeds through the establishment of in situ and ex situ conservation programs [21].

The current assessment of the variability level of the Venetian breeds revealed that the LAM and FOZ breeds, which had been subjected to a continuous loss of their genetic variability due to their limited population size, and therefore confronted with a high risk of disappearance, exhibited the highest expected and observed heterozygosities, the latest consanguinity level, and a considerable AR (Table 3). Thus, these findings highlighted the success of the conservation strategies applied to save these two big-sized breeds, representing the few remaining parts of the traditional transhumance system that are disappearing [47]. The considerable richness in private alleles revealed within FOZ and LAM breeds (33% and 29%, respectively) reflected the specific characteristics of these breeds, such as the high wool quality for FOZ [87] and the typical smoked meat, and the high rusticity of LAM breed, which is still maintained in a semi-wild state without the use of any housing throughout the year [21,87].

The genetic variability level of the medium-sized BRO breed is highlighted by its considerable allelic and gene diversity (Table 3). This multipurpose breed (meat, milk, and wool), keeping its traditional small flock farm breeding system, presented the highest richness in private alleles (38%), reflecting its specific characteristics. In fact, this breed, mainly reared for meat production, produced taller and fatter lambs with a smaller pelt proportion than the ALP bread reared in the same breeding system [47]. This characteristic can be associated with its more dairy vocation since its milk has been traditionally transformed to produce typical cheeses [21,47]. A previous genetic evaluation of Venetian breeds [21] revealed a lower allelic and gene diversity level and a higher consanguinity in this breed, which revealed an improvement in the breed’s genetic variability through the established conservation program.

The ALP breed, despite its decreasing population size in the last decade (from 3219 heads with 51 males and 3145 breeding females enrolled in herd books in 2012, to only 1923 animals with 91 males and 1573 breeding females enrolled in herd books in 2021 [10]), exhibited the highest allelic diversity, the lowest observed heterozygosity, and the highest homozygote excess coefficient among the four Venetian breeds (Table 3). Dalvit et al. [21] in 2009 revealed a close level of Ho and inbreeding coefficient but lower allelic and gene diversity, which indicates the improvement of the breed diversity level through the established conservation program. This small-sized breed, considered traditionally a multipurpose breed (meat, milk, and wool) well adapted to mountainous areas, is currently famous for the lamb production of excellent quality, especially if reared in organic farming conditions under wild or semi-wild management, guaranteeing this excellent meat quality [47,86]. The ALP’s specific meat quality, recently confirmed by the comparative analysis between the four Venetian breeds’ meat qualities conducted by Bittante et al. [47], explain its high level of private allelic richness (36%). In fact, ALP lambs benefit from Slow Food Praesidium certification and were elected one of the best 270 lambs in the world by the Veneto Region Slow Food Foundation. Its meat is sold to the best Venetian restaurants under the Foundation supervision [14,21,47].

Considering the Venetian breeds as the whole population (data not shown in Table 3), the assumed removal of ALP will reduce the overall diversity of this pool due to a loss of the between breeds diversity (−0.34%) and will increase it due to its internal diversity (+1.42%), thus obtaining a global gain of +1.08%. The removal of each remaining breed BRO, FOZ, or LAM will reduce this pool’s diversity by respectively −0.35%, −1.2% and −0.75%, due to the reduction of both within and between breed diversities. Dalvit et al. [21] in 2009, revealed a greater loss of genetic diversity after the removal of BRO, FOZ, and LAM of −1.06%, −1.64% and −1.07% respectively and a loss of −0.51% after the removal of ALP. Despite the conservation strategies established in the last decades in the Conservation Center of the experimental “Villiago” Mountain Farm and by the private breeders grouped under the Veneto breeders’ associations [14,87], the current genetic variability assessment revealed that the Venetian breeds situation remains critical. Consequently, the conservation decision of these Venetian local breeds already undergoing in situ and ex situ conservation programs should be continued despite the improvement of the situation, especially for the ALP breed.

The center Italian APP breed, a very old breed originating from the Appennine Mountain, is declared by the DAD-IS system as “Not at Risque Local Native breed,” with a noteworthy decrease in the last two decades from 150,000 animals in 1991, 14,335 in 2001 to only 8755 heads in 2021 [10]. The genetic diversity analysis revealed that APP exhibited the lowest allelic and genetic diversity levels and the highest inbreeding coefficient after the Venetian ALP breed. The APP breed is raised for meat vocation; however, no specific characters or meat quality were revealed in this center Italian breed, which can explain its lowest richness in private alleles within the Italian group. A similar genetic diversity level was revealed in APP [48,49,88]. Ceccobelli at al. [88] declared that the current APP breed resulted from crosses practiced in the last decades between non-selected ancestral APP populations well adapted to high altitude mountain pastures such as the Pagliarola, and selected meat breeds, mainly the Bergamasca. Based on Caballero and Toro’s method [33], the assumed removal of APP will increase the overall diversity of the analyzed breeds due to its internal diversity (+0.39%) and reduce it due to the loss of the between breeds diversity (−0.24%), obtaining thus a global genetic diversity gain of +0.15%. Accordingly, compared to the remaining analyzed breeds, APP would not be a priority for conservation programs. However, due to its notable population size reduction and the dilution of its ancestral gene pool by crossing with highly selected meet breeds, conservation strategies need to be established to preserve this breed.

#### 3.3.3. Diversity Contribution of the Private and Public Sheep Breeding Sectors

The comparison between the private and public management practices in both Mediterranean sides’ sheep breeding sectors was carried out based on the heterozygosity and consanguinity levels, and on the results of the Caballero and Toro approach applied to private and public flocks of each breed, as illustrated in Table 4. Relative conservation priorities were elaborated for each breed and for each case (private and public), if available. As mentioned previously, the Tunisian Public sector holds the three meat breeds BAR, QFO, and NTH and the unique dairy breed SS. This sector is managed by the (OEP) Office mandated by the Ministry of Agriculture to monitor sheep breeding improvement programs, which are part of sectorial animal genetic resources development strategies. These strategies focus on genetic improvement as an essential pillar development of the sheep breeding sector, basically the meat production capacities of the local breeds, through improving the morphological and genetic characteristics of the herds. These programs are followed by improving the herds’ environmental condition factors through identification programs, performance monitoring, artificial insemination, natural breeding, pedigree, genetic evaluation, and selection. Nevertheless, several problems in the identification method have been revealed by the OEP, which are related to the low rate of herd coverage and the difficulty of the technical and sanitary monitoring of the animals, especially concerning the private breeders, where the controlled herd’s rate does not exceed 15% [59,78].

The sampled public animals belong to the OTD flocks, which hold 56% of the public-controlled sheep, with 55,000 sheep heads and 69 controlled flocks. The major part of the OTD flocks consists of the fat-tailed BAR breed divided into two herd’s group: BAR with a red head (BTRO, 90%) and BAR with a black head (BTNO, 10%); the remaining flocks belong to the QFO (7 flocks), the NTH (6 flocks), and the SS dairy breeds (2 flocks) [19,89]. Besides the general management recommendations enrolled in the OEP, the OTD management strategy is based on extensive management for the BAR and QFO breeds and semi-extensive for the NTH and SS breeds, based on the establishment of annual contracts with shepherds before the lambing season. They are responsible for the adopted management practices application, with continual (monthly and annual) inventories and internal/external auditor evaluation systems [89].

The comparison between Tunisian public and private flocks’ genetic parameters results (Table 4) revealed no clear difference in the gene diversity levels (He) between the two sectors, despite the management strategies applied by the public sector for half a century for all the analyzed breeds. The Ho was lower than expected for the three breeds, except for the BTNO flock, which presented very close values (He = 0.800 and Ho = 0.799). This flock exhibited the lowest inbreeding coefficient (F_IS_ = 0.012), thus reflecting the random mating within the BTNO population, and its successful management was not negatively influenced by the selection pressure. This worthy genetic diversity level is reflected by the high production performance of this population, in addition to its superior rusticity and adaptation traits [19,89]. The revealed difference between the two BAR flocks BTRO and BTNO (F_IS_ = 0.054 and 0.012; Ho = 0.755 and 0.799, respectively) could be explained by the difference in the management practices applied by the OTD services between these two flocks. The population size of the BTRO (16,868 UF, 90%) is higher than that of the BTNO (3607 UF, 10%), which engenders more vigorous management of this last group in the paternity control. In fact, under the control of the OEP Office, best lamb selection is monitored based on the recorded information and the performance characters from the totality of Tunisian public farms, mainly from the OTD flocks (a total of 69 controlled flocks). The unselected lambs from the collected and indexed OTD lambs, after performance and sanitary controls, are dispatched randomly on the OTD farms and can return to their origin flocks. The selected lambs were kept and reared in the OEP genitors’ center and then dispatched randomly on public farms. Consequently, genitors might return to their original flocks due to the rotation of sires practiced by the office, especially for the BTRO-abundant flocks from which the majority of the selected BTR rams originated [19]. For the BTNO, when selected lambs are not sufficiently available from the BTNO flocks, which is often the case, rams with high performance were purchased from other OEP-controlled farms, which increases the genetic variability within the BTNO population (OTD, personal communication) [19]. These purposes were justified by the difference in the internal diversity of these two public BAR groups revealed by Caballero and Toro’s approach [33] (data not shown in Table 4), which is positive (+0.015%) in BTNO population and negative in BTRO (−0.068%). The loss of genetic diversity resulting from the removal of BTRO (−0.068%) is resulted mainly from a loss of within-population diversity of −0.057% and between-population diversity of −0.011% (data not shown in Table 4). Otherwise, considering the BAR breed as the whole data, the results of Caballero and Toro’s approach [33] (values with * in Table 4) showed a loss of global genetic diversity in both private and public (OTD) cases after removing BTR flocks; however, no genetic loss was observed in the cases of private or public BTN flock removal. These findings further highlighted the genetic superiority of the BTN, which is supported by the OTD and the private owners’ declarations considering the BTN sheep are more rustic. For the private sector, the removal of the two main variants of the BAR breed (BTN) and BTR would produce a substantial increase in diversity, indicating that no conservation decision is needed to preserve the genetic diversity within this breed. Despite the lack of a clear breeding improvement program in this sheep breeding sector based mainly on traditional management, the private BAR flocks BTR and BTN exhibited a high overall variability level, illustrated by a high heterozygosity (Ho = 0.739 and 0.762, respectively) and low inbreeding coefficient (F_IS_ = 0.087 and 0.047, respectively). This could be explained by the mating practices in this breed imposing a paternity control system due to shepherd assistance during mating, as previously explained. For the public flocks of the BAR breed, it is worth mentioning that the risk threatening this breed manifested by the dilution and absorption of its gene pool by anarchic crossing with the QFO breed is avoided. Standard breed characteristics have been maintained through public management practices, even though animals with a very big fat-tail, which is the specific rustic characteristic of the breed considered as body reserve mobilized under harsh conditions, have usually been reformed from the OTD herds [19]. Furthermore, a linear decrease in the average weight and weight gain growth performance of BAR lambs (average of 8 kg) has been reported since the beginning of the improvement program in the sixties [19,90,91]. This can be explained by several weaknesses revealed in the current BAR breeding strategy applied during a half-century, resumed in the lack of clear breeding objectives and unclearness of involved traits and their economic weights, and the low number of selected rams per year, which is not proportional to the large BAR’s population size [90]. Conservation strategies accounting for the high within-breed diversity may tend to give priority to the breed with the largest population size exhibiting the highest internal diversity [33], which is the case with the BAR. Therefore, revision of the current national management strategy of the BAR to develop more coherent and sustainable strategies is an urgent need. A clear in situ conservation program is needed to safeguard the rusticity characteristics through keeping the breed in the marginal area and low input production system wherein it is well adapted; valorizing its typical meat; and valorizing the significant quantity of the caudal fat (reaching 7 kg per animal) that can be transformed into agro-fuel, such as has been done for pork and duck fat. The widening of future strategies to include the private sector and small breeders is necessary since these latter constitute the major part (64%) of sheep owners.

Regarding the NTH breed, both public and private sectors’ animals presented quite similar large gene diversity and low inbreeding levels. This situation is explained by the similar expert management practices applied by the OTD and private owners, who reared the breed under a semi-intensive breeding system in the Northwestern big farms. Caballero and Toro’s approach [33] revealed that both private and public NTH flocks need conservation decisions since their assumed removal will decrease the overall diversity by −0.13% and −0.06% for NTH and NTHO, respectively.

The SS dairy breed management appeared more efficient in the private sector than in the public one, which included lower Ho, He, and higher F_IS_ values (Table 4). The improved situation of the private SS sector results from the creation of the SS breed association by local breeders to rescue the breed after its drastic decrease. The valorization of the SS’s milk through doubling its price in one year, as well as the cooperation between the SS association and national and international research institutions to reduce the inbreeding level, allowed the breed to regain its interest [70] and overcome the bottleneck effect [25]. Despite this progress, Caballero and Toro’s approach [33] revealed that both SS’s breeding sectors are a priority for conservation decisions, even for the private flocks, whose removal will result in a bigger loss of the GD (−0.38% versus −0.11%).

As illustrated in Table 4, the private QFO flock exhibited a similar level of expected and observed heterozygosities to the public flock (QFOO). However, a higher consanguinity level was revealed in the private flocks. Caballero and Toro’s method [33] revealed that the assumed removal of the private QFO subpopulation will reduce the overall private and public sheep pool diversity (−0.28%) due to the loss of its within-breed diversity (−0.64%), since its mean distance from the remaining subpopulation will increase the overall pool diversity (+0.36%, data not shown in Table 4). A conservation decision would be needed for this private sector, currently holding about 1,330,100 productive ewes (35%) of the total Tunisian sheep resources [78].

These comparative analyses confirm that considerable genetic variability exists in both the public and private sectors’ sheep resources. Despite the establishment of genetic improvement programs in the public sector for more than half a century, no notable improvement is detected at the genomic level explored by microsatellite polymorphism, which is reflected by the lack of productivity improvement. The Tunisian sheep available genetic variability is the raw material that can be used to make the breeds more efficient in using available feed and water resources, even if they are scarce [90]. This latter study revealed that the poor production ability of Tunisian sheep breeds is linked to the lack of coherent breeding strategies and breeders’ organizations [90]. An urgent need for firmly establishing these two conditions is indispensable for Tunisian sheep resource improvement and preservation.

The comparison of the genetic variability level between the private and the institutional (public) endangered Venetian sheep breeds revealed a higher gene diversity level (He) in the private sector, except for the LAM breed, which presents higher He and Ho within the public flocks. The inbreeding level was higher in private flocks, especially for the ALP and BRO breeds (respectively 0.139 and 0.109 in private flocks versus 0.025 and 0.035 in institutional ones). This low inbreeding level is explained by the management strategy applied in the public sector monitored by the “Villiago” Conservation Center, which aimed to preserve a stock number of breeds presenting the standard functional and morphological characteristics through adequate mating strategies enabling the inbreeding level reduction within each breed [14,86]. Both in situ and ex situ conservation programs have been adopted by the public breeding sector [14,47,86]. In situ conservation management strategies are adopted by the private sector and monitored by breeders’ associations holding smallholders, mainly with part-time farmers [86]. Considering sampled animals from both private and institutional farms as the whole data set, the total contribution of each sector to genetic diversity (Loss/Gain), after the hypothetical removal of private or institutional herds, was envisaged as proposed by Caballero and Toro’s approach [33] (Table 4). Results revealed that for the “Villiago” Conservation Center, the situation of the FOZ and LAM breeds still needs conservation priorities, especially for LAM since its removal resulted in a loss of −1.38% of Venetian genetic diversity pool versus a loss of −0.17% for FOZ. The institutional flocks of ALP and BRO seem to have restored their genetic diversity, since their removal modestly contributed to the overall Venetian sheep diversity with a global positive balance of +0.05% and +0.55%, respectively, resulting from within and between population diversities. The situation of the private sector is more critical since the 3 breeds ALP, BRO, and FOZ still need conservation priority decisions, as revealed by Caballero and Toro’s approach [33]. In fact, the removal of these three breeds’ private herds will produce a large decrease in the overall Venetian sheep diversity of −4.92%, −5.76% and −6.08%, respectively, in the remaining pool (Table 4). Supplementary efforts must be proved by private Venetian sheep breeders reunited under the Veneto Regional Breeders Association (ARAV). Continual efforts are still being made to improve the competitiveness of these endangered native breeds, the valorization of their local products, and their linkage with mountain agro-ecosystems [47,86]. Recently, a smartphone application has been elaborated and considered an efficient communication tool to avoid inbreeding during mating plans through the exchange of the most genetically distant rams between farmers. Furthermore, this application allowed the exchange of updated farm information and activities, such as agro-tourism, territorial marketing strategies, and the contribution of their grazing activities to the environment and society [86]. The conversion of ordinary farming to organic farming is strongly encouraged to further valorize Venetian sheep resources; even financial aid to support smallholders is indispensable to generalize this trend [86].

### 3.4. Breed Relationships and Differentiation

#### 3.4.1. Factorial Correspondence Analysis (FCA)

**Figure 1:**
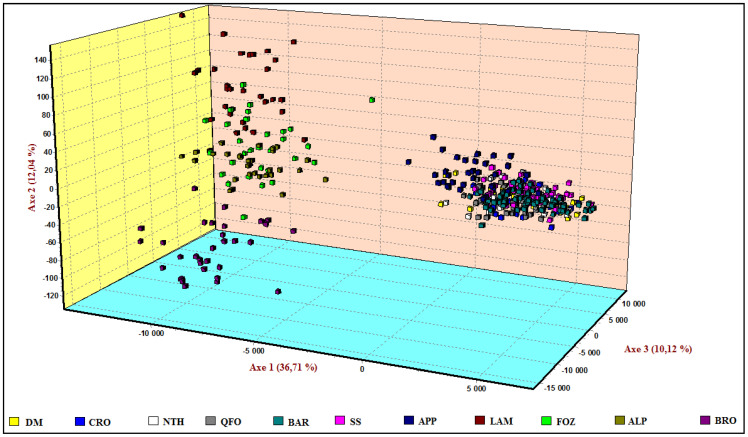
Spatial representation of the Mediterranean-analyzed native sheep breeds as defined by factorial correspondence analysis. Tunisian breeds: Barbarine (BAR), Queue Fine de l’Ouest (QFO), cross-bred BAR × QFO (CRO), D’man (DM), Sicilo-Sarde (SS), Noire de Thibar (NTH); Venetian breeds: Alpagota (ALP), Brogna (BRO), Foza (FOZ), and Lamon (LAM); Central Italian breed: Appenninica (APP).

The Factorial Analysis of Correspondence was performed, including both Mediterranean sides’ local sheep breeds, based on all microsatellite loci’ allele frequencies (Figure 1). The first three components explained 58.87% of the total variation, 36.61% of which are explained by Axis 1, which clearly separates the group of the Venetian breeds from the very close group formed by the Center Italian APP and the Tunisian breeds. This genetic closeness of the APP and the Tunisian breeds supported the morphological similarity discovered between the APP and QFO breeds, which was the main reason for including APP in these investigations. Axis 2 shows 12.04% of the variance and separates the four Venetian breeds group that appeared more differentiated than the Tunisian breeds-APP group, especially for the BRO and LAM breeds, which are clearly separated from the FOZ and ALP individuals.

#### 3.4.2. POSA Distances and Neighbor Network Analysis

**Figure 2:**
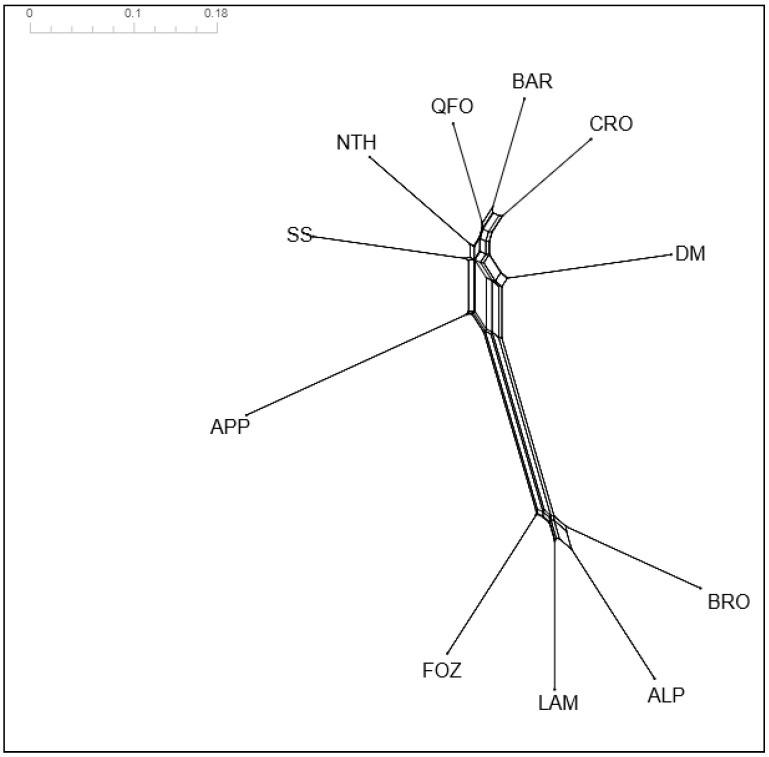
Neighbor network representation of the Proportion of Shared Allele (POSA) distances between the analyzed native breeds; Tunisian breeds: Barbarine (BAR), Queue Fine de l’Ouest (QFO), cross-bred BAR × QFO (CRO), D’man (DM), Sicilo-Sarde (SS), Noire de Thibar (NTH); Venetian breeds: Alpagota (ALP), Brogna (BRO), Foza (FOZ) and Lamon (LAM); Central Italian breed: Appenninica (APP).

The representation of the POSA distances through a neighbor-net graphic (Figure 2) showed that the APP center Italian breed is grouped in the same branch as the Tunisia native breeds and is especially closer to the SS and NTH breeds exhibiting European (South Italian and French, respectively) gene pools, which highlight their between shared alleles. The NTH breed is close to the QFO-BAR-CRO sub-branch, illustrating the ancestral relationship between the NTH and its ancestor, the QFO. This last sub-branch illustrates well the ancestral relationship between the BAR, the QFO, and their crossbred CRO. The DM breed, belonging to a unique sub-branch, is the closest south Mediterranean breed to the Venetian breeds. Within the Venetian breeds group, two sub-groups are shown: the ALP and BRO sub-branch grouping the two medium-sized Venetian breeds are separated from the sub-branches of the large-sized and transhumant LAM and FOZ breeds. The geographic, genetic, and morphological (carcass conformation) closeness of these two large-sized breed has been evidenced [21,47]. The differentiation of the FOZ sub-branch would be related to the distinguished wool quality of this breed [87], exhibiting 33% of the specific allele.

The phylogenetic relatedness of the Tunisian breeds and the analyzed native Italian breeds was recently detailed by Ben Sassi-Zaidy et al. [13]. A strong and very ancient gene flow is evidenced between the Tunisian and the Italian analyzed breeds, which traces back to the early Neolithic between the Venetian and the Tunisian (Maghrebian) native breeds, and to the Carthaginian and Roman periods. It continued into the medieval period between the center Italian APP and the Tunisian breeds, especially between APP and QFO, which share the highest gene flow [13]. Ceccobelli et al. [88] highlighted an ethnological relationship between the Pagliarola population belonging to the Appennine sheep group and an Arabian salted meat sheep, which is remote to the Arabian presence in the Italian peninsula. These findings emphasized the genetic relatedness between APP and Tunisian sheep resources.

#### 3.4.3. Genetic Structure Analysis

**Figure 3:**
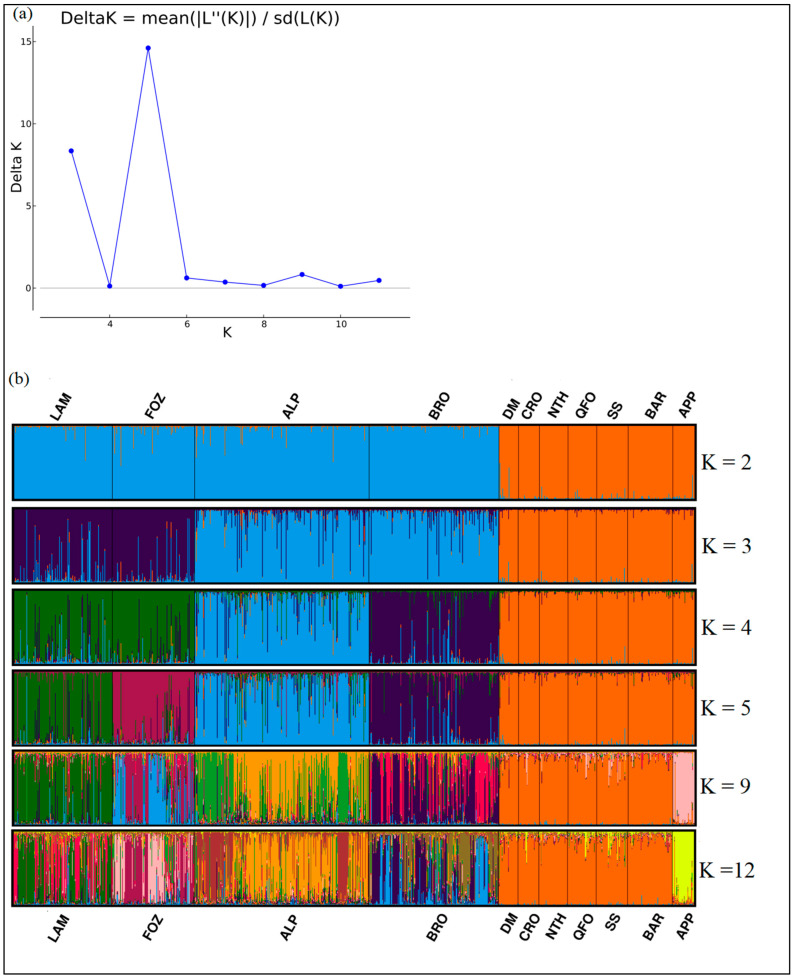
(**a**) Delta K values obtained with the STRUCTURE HARVESTER. (**b**) Estimated population structure of the analyzed breeds represented as bar plot depicting individual membership proportions for each cluster; Venetian breeds: Alpagota (ALP), Brogna (BRO), Foza (FOZ) and Lamon (LAM); Central Italian breed: Appenninica (APP); Tunisian breeds: Barbarine (BAR), Sicilo-Sarde (SS), Queue Fine de l’Ouest (QFO), Noire de Thibar (NTH), cross bred BAR × QFO (CRO), D’man (DM).

The genetic structure of the analyzed Tunisian and Italian breeds was investigated using the Bayesian approach with K number of expected clusters ranging from 2 to 12. The optimal number of ancestral populations was K = 5 (Figure 3a). At K = 2, the ancestral relatedness between the Venetian breeds group on the one hand, and the totality of the Tunisian breeds and the Center Italian APP breed on the other hand is illustrated in Figure 3b. The strong uniqueness of this last group was maintained until K = 9, in which the distinctness between APP and Tunisian breeds occurred. At K = 3, the Venetian breeds were divided into 2 clusters: the medium-sized ALP and BRO cluster and the cluster of the large-sized and transhumant LAM and FOZ breeds, which is maintained at K = 4. These clustering results corroborated the neighbor network analysis. At the best-assumed cluster (K = 5), the Venetian breeds were assigned into 4 distinguished clusters illustrating their current classification. For further increased K values, further admixtures were observed within the Venetian breed clusters, highlighting their within-breed fragmentation [21]. The Tunisian breeds maintained their clusterization as a unique group. Deeper structure analysis of the APP-Tunisian breeds’ group was recently envisaged [13] highlighting this strong relatedness already detailed in the neighbor network analysis.

## 4. Conclusions

In this study, we assessed the genetic diversity of all locally adapted Tunisian sheep breeds and five native Italian breeds in both the private and public breeding sectors based on the microsatellite genotyping technique. The obtained results give deeper insights into the genetic diversity level of the analyzed breeds, which appeared more genetically diversified in the Southern Mediterranean rive, and slightly more efficiently managed in the public breeding sector than the private one in both countries. As a general outcome of this bi-dimensional comparison, at the genetic diversity and breeding management axes of both Tunisian and Italian sheep genetic resources situations, we revealed that the Tunisian sheep sector possesses the desired genetic diversity base and a considerable population size, but it still needs efficient and coherent breeding and conservation strategies. The Italian side, exhibiting currently efficient conservation and breeding strategies and the necessary financial tools, nevertheless suffers from the drastic reduction in the size of the native breeds’ population over the last decades, which will be hard to reverse.

Adequate conservation decisions are recommended to conserve Tunisian sheep resources, exhibiting a significant population size and genetic diversity that can be oriented to short-term and long-term conservation strategies. In situ conservation strategies involving breeders, who should be grouped under breed associations, seem urgently needed to ensure the breeds’ valorization and the avoidance of inbred mating and breed’s erosion.

Concerning Italian native sheep breeds, stopping the current decrease in the APP breed population size and preserving or improving its genetic variability level are urgently needed. Further valorization of endangered Venetian native breeds is still needed, despite the current efforts. Promoting agro-tourism and the conversion of ordinary breeding to organic farming seem to be successful tendencies.

Complementarity of these two different situations under developing and developed countries’ conditions helps to build efficient sustainable sheep breeding strategies based on the conservation of within and between breed genetic diversity to handle future challenges in sheep breeding and food security.

## Figures and Tables

**Table 1:** Information about the sampled Tunisian and Italian locally adapted sheep breeds: Tunisian breeds: Barbarine (BAR), Queue Fine de l’Ouest (QFO), cross-bred BAR × QFO (CRO), D’man (DM), Sicilo-Sarde (SS), Noire de Thibar (NTH); Venetian breeds: Alpagota (ALP), Brogna (BRO), Foza (FOZ) and Lamon (LAM); Central Italian breed: Appenninica (APP).

Country	Breed	Classification/Origin [20,21]	Vocation/Environment [20,21]	Risk Status [5]	Conservation Program [5]
Tunisia	BAR	Local rustic fat-tailed/very old (Phoenician)	Typical flavored Meat/well adapted to all climate stage (Humid to Saharan)	Not at Risk	In Situ
	QFO	Local (Algerian Ouled Djellel)	Meat/Western High altitude (Northern and Eastern pervasive)	Not at Risk	No
	CRO	Recent Crossbred (BAR × QFO)	Meat/created in Center (North and East spreading	-	No
	NTH	Local (Last century)	Meat/Sub humid to Semi-arid	Not at Risk	No
	SS	Local (Last century)	Traditional sheese/Sub humid	Unknown	No
	DM	Maghrebian/(Oasis Morocco)	Meat and prolificacy/Saharan	-	No
Italy	ALP	Local	Typical Meat/Alpago mountains	At Risk endangered	In Situ
	BRO	Local	Typical cheeses/Lissini mountains	At Risk endangered	In Situ
	FOZ	Local	Wool/Transhumance in Asiago mountains	At Risk Critical	In Situ
	LAM	Local	Typical smoked meat/Semi-wild state: migration between hill, plain and mountains	At Risk endangered	In Situ
	APP	Local	Meat/Central Italy	Not at Risk	In Situ

**Table 2:** Characteristics of the microsatellite loci used to genotype the sampled Tunisian and Italian locally adapted sheep breeds.

Microsatellites			Tunisian Breeds			Italian Breeds			Whole Data		
Locus	Size	Ch	TNA	AR	PIC	TNA	AR	PIC	TNA	AR	PIC
Inra023	195–221	1	14	11.46	0.89	16	8.31	0.84	17	10.25	0.87
Inra063	168–208	14	20	12.71	0.84	25	7.82	0.79	28	10.40	0.82
OarCP49	71–137	17	29	18.02	0.90	24	8.41	0.79	31	13.34	0.85
OarFCB304	145–201	19	21	11.70	0.81	18	6.43	0.69	24	8.96	0.76
OarFCB20	85–121	2	17	11.94	0.87	17	7.42	0.8	19	10.26	0.84
MAF65	113–139	15	13	8.53	0.76	18	5.81	0.75	21	7.75	0.75
ILST087	134–184	6	25	15.67	0.90	23	9.33	0.85	26	13.17	0.88
OarAE119	145–185	19	14	10.53	0.81	20	4.82	0.66	20	9.51	0.82
MCM527	164–190	5	13	8.77	0.81	15	6.34	0.76	17	7.89	0.78
MAF214	182–262	16	15	6.97	0.62	36	5.71	0.6	38	6.96	0.61
OarAE129	135–165	5	8	5.67	0.60	15	7.92	0.84	16	5.26	0.63
OarCP34	93–117	3	10	6.55	0.77	18	6.44	0.78	18	6.62	0.77
OarAE54	120–152	25	16	10.97	0.79	19	9.31	0.86	19	10.44	0.82
TGLA	125–163	12	15	10.78	0.85	14	7.72	0.82	16	9.67	0.84
URB	159–211	13	22	10.95	0.84	20	9.91	0.88	24	9.93	0.86
CSRD	208–262	14	26	10.88	0.85	24	9.11	0.83	28	9.74	0.84
HSC	260–296	20	19	9.86	0.86	19	8.92	0.85	21	9.33	0.85
Average	-	-	17.47	10.70	0.810	20.06	7.63	0.788	22.53	9.38	0.80
SD	-	-	5.75	3.07	0.086	5.29	1.49	0.076	6.07	2.09	0.08

Ch, chromosome; NA, number of alleles; AR, allelic richness; PIC, polymorphic information component, SD, stander deviation.

**Table 3:** Genetic diversity estimates in Tunisian-Italian corridor’s local sheep breeds. (N), number of analyzed samples; (AR), allelic richness obtained with the rarefaction method; (P_AR_), allelic richness of private alleles; (He), expected (Ho), observed heterozygosity, within-breed heterozygote deficiency (F_IS_); (HWE), number of loci deviated from the Hardy–Weinberg equilibrium; GD (Loss/Gain), proportional loss or gain in global genetic diversity after hypothetically removing each breed and relative conservation decision.

Breed	N	AR	P_AR_	He	Ho	F_IS_	HWE	GD Loss (−)/Gain (+)/Conservation Decision *
BAR	64	10.05 ± 3.51	0.34	0.83 ± 0.10	0.75 ± 0.18	0.073 ± 0.062	1 ***	+0.227/No
QFO	41	10.40 ± 3.05	0.30	0.82 ± 0.03	0.74 ± 0.14	0.101 ± 0.143	2 ***	+0.119/No
CRO	30	09.73 ± 3.01	0.25	0.80 ± 0.10	0.68 ± 0.17	0.139 ± 0.192	2 ***	+0.213/No
NTH	41	09.29 ± 2.34	0.16	0.82 ± 0.04	0.75 ± 0.15	0.071 ± 0.121	1 ***	+0.123/No
SS	45	10.05 ± 2.95	0.38	0.82 ± 0.02	0.73 ± 0.13	0.102 ± 0.103	2 ***	−0.064/Cons
DM	28	10.38 ± 3.40	0.45	0.82 ± 0.01	0.72 ± 0.17	0.123 ± 0.138	1 ***	−0.041/Cons
ALP	250	09.01 ± 1.71	0.36	0.77 ± 0.11	0.67 ± 0.16	0.134 ± 0.132	12 ***	−0.280/Cons
BRO	186	08.97 ± 1.72	0.38	0.79 ± 0.08	0.70 ± 0.10	0.109 ± 0.112	8 ***	−0.372/Cons
FOZ	118	08.91 ± 1.83	0.33	0.81 ± 0.09	0.72 ± 0.15	0.107 ± 0.150	4 ***	−0.493/Cons
LAM	141	09.01 ± 1.73	0.29	0.80 ± 0.08	0.72 ± 0.11	0.101 ± 0.101	6 ***	−0.470/Cons
APP	31	07.56 ± 2.41	0.21	0.76 ± 0.08	0.66 ± 0.14	0.119 ± 0.184	3 ***	+0.151/No

*** *p* < 0.001; * Cons/No, Conservation decision based on Caballero and Toro’s approach [33]: +, assessed breed would not be preferred for conservation (No); −, assessed breed would be preferred for conservation (Cons); Tunisian breeds: Barbarine (BAR), Queue Fine de l’Ouest (QFO), cross-bred BAR × QFO (CRO), D’man (DM), Sicilo-Sarde (SS), Noire de Thibar (NTH); Venetian breeds: Alpagota (ALP), Brogna (BRO), Foza (FOZ) and Lamon (LAM); Central Italian breed: Appenninica (APP).

**Table 4:** Comparison of genetic diversity levels between private and public/institutional breeding systems and conservation decisions according to Caballero and Toro’s approach.

Sheep Populations			N	He	Ho	F_IS_	Loss (−)/Gain (+) of GD	C&T Conservation Decision
Tunisian sheep breeds	NTH	NTH	26	0.800 ± 0.091	0.750 ± 0.158	0.063	−0.130	Cons
		NTHO	15	0.800 ± 0.101	0.747 ± 0.172	0.068	−0.056	Cons
	SS	SS	30	0.812 ± 0.097	0.738 ± 0.133	0.092	−0.384	Cons
		SSO	15	0.808 ± 0.123	0.706 ± 0.198	0.129	−0.110	Cons
	QFO	QFO	34	0.828 ± 0.068	0.741 ± 0.134	0.107	−0.284	Con
		QFOO	15	0.795 ± 0.149	0.738 ± 0.236	0.076	0.017	No
	BTR	BTR	31	0.804 ± 0.094	0.739 ± 0.137	0.083	0.112/−0.034 *	No/Cons *
		BTRO	17	0.804 ± 0.090	0.755 ± 0.156	0.054	0.012/−0.068 *	No/Cons *
	BTN	BTN	31	0.798 ± 0.103	0.762 ± 0.152	0.047	0.161/0.251 *	No
		BTNO	15	0.800 ± 0.109	0.799 ± 0.161	0.012	0.108/0.056 *	No
Venetian sheep breeds	ALP	ALPP	186	0.773 ± 0.113	0.662 ± 0.162	0.139	−4.922	Cons
		ALPI	64	0.717 ± 0.158	0.704 ± 0.190	0.025	0.051	No
	BRO	BROP	128	0.793 ± 0.076	0.696 ± 0.085	0.109	−5.763	Cons
		BROI	58	0.740 ± 0.094	0.712 ± 0.140	0.035	0.549	No
	FOZ	FOZP	76	0.801 ± 0.086	0.730 ± 0.176	0.087	−6.080	Cons
		FOZI	42	0.760 ± 0.097	0.707 ± 0.141	0.073	−0.167	Cons
	LAM	LAMP	100	0.740 ± 0.094	0.712 ± 0.140	0.035	0.549	No
		LAMI	41	0.784 ± 0.063	0.747 ± 0.131	0.036	−1.377	Cons

Tunisian breeds: Noire de Thibar (NTH), Sicilo-Sarde (SS), Queue Fine de l’Ouest (QFO), Barbarine flock with Red Head (BTR), Barbarine flock with Black Head (BTN); Venetian breeds: Alpagota (ALP), Brogna (BRO), Foza (FOZ), and Lamon (LAM); “O” at the end of Tunisian breed name: belonging to the Tunisian OTD public farms; “P” at the end of the Venetian breed name: private Venetian farms; “I” at the end of Venetian breed name: Institutional/Public Venetian farms; Cons/No, Conservation decision based on Caballero and Toro’s approach [33]: +, assessed breed would not be preferred for conservation (No); −, assessed breed would be preferred for conservation (Cons); * Gain/loss of GD considering the BAR breed herds as whole data.

## Data Availability

Not applicable.
